# Effect of Dope Flow Rate and Post-Treatment on the Morphology, Permeation and Metal Ion Rejection from PES/LiBr-Based UF Hollow Fiber Membranes

**DOI:** 10.3390/membranes12030305

**Published:** 2022-03-09

**Authors:** Muhammad Irfan, Masooma Irfan, Ani Idris, Abdullah Saad Alsubaie, Khaled H. Mahmoud, Noordin Mohd Yusof, Nawshad Muhammad

**Affiliations:** 1 Centre for Environmental Sustainability and Water Security (IPASA), School of Chemical and Energy Engineering, Universiti Teknologi Malaysia, Johor Bahru 81310, Malaysia; irfanutm@gmail.com; 2 Interdisciplinary Research Centre in Biomedical Materials, COMSATS University Islamabad, Lahore Campus, Defense Road, Off Raiwind Road, Lahore 54000, Pakistan; 3 Department of Chemistry, COMSATS University Islamabad, Lahore Campus, Defense Road, Off Raiwind Road, Lahore 54000, Pakistan; 4 Department of Physics, College of Khurma University College, Taif University, P.O. Box 11099, Taif 21944, Saudi Arabia; asubaie@tu.edu.sa (A.S.A.); k.hussein@tu.edu.sa (K.H.M.); 5 School of Mechanical Engineering, Faculty of Engineering, Universiti Teknologi Malaysia, Johor Bahru 81310, Malaysia; noordin@utm.my; 6 Department of Dental Materials, Institute of Basic Medical Science, Khyber Medical, University Peshawar, Peshawar 25100, Pakistan; nawshadbnu@gmail.com

**Keywords:** dope extrusion, PES, lake water, salt, hollow fiber

## Abstract

This study investigated the influence of dope extrusion rate (DER) and post-treatment effect on the morphology, permeation, and metal ion rejection by polyethersulfone/lithium bromide (PES/LiBr)-based hollow fiber (HF) membranes. HF fibers were spun with 2.25, 2.5, and 3.1 ratios of DER to bore fluid rate (BFR), wherein DER varied from 11.35, 12.5, to 15.6 mL/min with a fixed BFR (5 mL/min). Molecular weight cutoff (MWCO), pore size, water flux, and flux recovery ratio were determined, whereas lake water was used to observe the rejection rate of dissolved metallic ions. Results showed that with the increase of the DER wall thickness (WT), HFs increased from 401.5 to 419.5 um, and furthermore by the post-treatments up to 548.2 um, as confirmed by field emission scanning electron microscope (FESEM) analysis. Moreover, MWCO, pore size, and the pure water permeation (PWP) of the HF membranes decreased, while the separation performance for polyethylene glycol (PEG) solute increased with increasing DER. Post-treated HFs from 11.35 mL/min of DER showed 93.8% of MWCO value with up to 90% and 70% rejection of the arsenic and chromium metallic ions, respectively, in comparison with all other formulated HFs.

## 1. Introduction

In recent years, polymeric membranes have gained wide acceptance because of their performance, reliability, flexibility, and cost competitiveness. During the last two decades, a number of studies have been reported to address the rheological factors in the membrane fabrication process that mainly focused on HF compositions, temperature of dope solution and external/internal coagulation bath, shear stress within spinneret, and a relative exchange rate between the solvent and polymer solution at the inner and outer surfaces of the HFs membranes. However, no work has been published on the comparison of DER to BFR with post-treatment effect as per our literature knowledge. However, recently, research has been intensified on the effect of DER, causing shear stress within the spinneret because it is observed that the dope rheology has a very important role in the course of HF fabrication in order to obtain desired properties [1,2,3,4].

When the pressurized dope solution is subjected in the spinneret during HFs spinning, shear stress is induced within the cavity of spinneret. The stress is higher on the wall than the center. Consequently, these stresses may affect molecular orientation and relaxation at the outer surface of the HFs [5]. Generally, shear rate is increased with rise in the DER that will cause the molecules of dope solution to pack nearer to each other, leading to a tighter outer skin structure of HF. This compact and tight packing of HFs result in higher rejection and lower flux. Chung and co-workers investigated the effect of shear stress within the spinneret, finding that high shear lowered the water permeation but increased the selectivity, owing to compact molecular orientation inside the fibers [6].

Ismail et al. [5] studied the effect of varied DER from 2.0 to 4.0 cm^3^/min to 0.5 mL/min increments and fabricated the HFs by using polyethersulfone/N-methylpyrrolidone (PES/NMP) and PEG in the formulation with a mixture of potassium acetate and water as a bore fluid liquid. The authors concluded that the outer skin layer of the membranes became noticeably thicker with an increasing dope extrusion rate that decreased the water permeation and increased the separation performance. In another study, Kusworo et al. [7] examined the outcome of shear rate on molecular orientation of HFs, used for gas separation and reverse osmosis. The authors proposed that high-sheared HFs membrane had better performance for the rejection of reverse osmosis application and selectivity of particular gas. Nevertheless, numerous researchers also studied the effect of DER for selectivity of gas separation membrane [5,6,7], but none of these studies were focused on comparing the DER and BFR with the rejection of metallic ions from water.

Thakur et al. fabricated polyacrylonitrile a polymer-based HF membrane whose polymer and water flow rate were 3 g/min and 20 mL/min, respectively. FESEM results showed that the resulting HF contained outer and inner diameters of 1.3 mm and 0.7 mm, respectively [8]. Tang et al. examined the different concentrations (13, 15, and 17 wt. %) of dope solution with DER 25.1 mL/min (0.4 MPa) and tap water as bore liquid with pressure of 0.1 MPa to fabricated HF membrane. The authors concluded that different weight percentages of dope solution exerted different pressures on the spinneret, thus leading to variable structure. The outer and inner layers of HF became thicker with the enhancement of polymer solution, leading to change in morphology from finger-like projection to sponge structures [9].

Liu et al. spun polyurethane-based HFs and post-treated them with hot-air treatment. The authors concluded that both skin layers of HF became dense from heat treatment. The same results were reported by Tsai et al. [10,11]. Ani et al. fabricated PES/LiBr HF membranes and post-treated them with a hot water bath. The authors examined that post-treatment removes the loosely attached composition particles that fixed the formulation, swelled the membrane, and enhanced the pore size [12].

To the best of our knowledge, a systematic study of the effect of DER to the inner bore fluid rate (BFR) within a spinneret for the fabrication of HF membrane and morphology, permeability, and separation of metallic ions from lake water has not been reported. Further, different post-treatments have led to different results, which mainly depend on membrane application. Therefore, the main objective of this work was focused on the above, and HFs were spun using the best formulation as reported by Idris et al. (mentioned in Section 2.2) [13]. Three types of HFs (M1, M2, and M3) were fabricated with 2.25, 2.5, and 3.0 ratios of DER to BFR, respectively (details are mentioned in Table 1). According to Barzin et al. [14], post-treatment affects the performances of HFs, and therefore all three fabricated fibers were also post-treated (named as M1-P, M2-P, and M3-P) a in hot water bath. Then, the morphology of the post-treated and non-post-treated HFs was studied via FESEM, and we evaluated their flux rate, rejection rate, and pore size. The lake water was used as the incoming feed, and the metallic ion rejection was quantitatively measured with turbidity and color analysis.

## 2. Materials and Methods

PES (Ultrason E6020P; molecular weight = 58,000 g/mol) were provided by BASF Co. (Ludwigshafen, Germany). Analytical-grade dimethylforamide (HCON(CH_3_)_2_; molar mass = 73.10 g/mol) was purchased from Merck (Darmstadt, Germany), analytical-grade anhydrous lithium bromide (LiBr) (molecular weight = 86.85 Dalton) was obtained from Sigma, and *polyethylene glycol* (PEG) (MW = 600, 1K, 3K, 6K, and 10K Dalton) was obtained from Fluka. Tap water was used in the coagulation bath.

### 2.1. Preparation of Dope Solution 

Microwave technique was used for the preparation of dope solution as described elsewhere [13]. Briefly, in a round bottle flask, 20 wt. % PES and 3% LiBr were blended in DMF solvent for 20 min in a microwave, and then the solution was further homogenized by stirring rod for 30 min without heating. The dope solution was stored in a 1 L bottle for 48 h, and air bubbles were removed by gravity.

### 2.2. Spinning of Hollow Fiber Membranes

**Figure 1:**
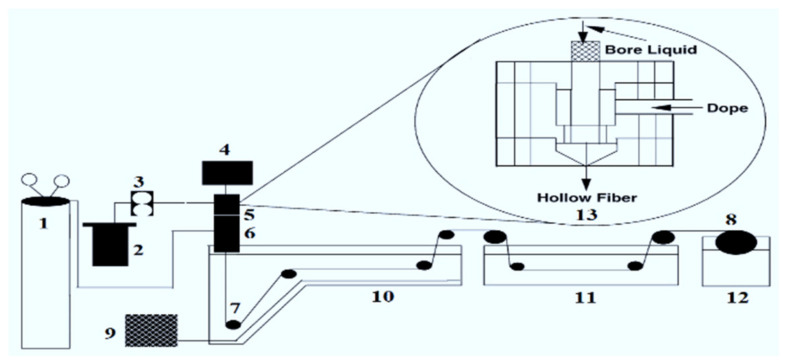
Schematic diagram of hollow fiber spinning system: (1) nitrogen cylinder; (2) dope reservoir; (3) gear pump; (4) on-line filter, 7 mm; (4) syringe pump; (5) spinneret; (6) forced convective tube; (7) roller; (8) wind-up drum; (9) refrigeration/heating unit; (10) coagulation bath; (11) washing/treatment bath; (12) wind-up bath; (13) schematic spinneret [5].

Asymmetric HF ultrafiltration membranes were fabricated via dry–wet phase inversion spinning process using tap water as an external coagulant bath, as described by Figure 1 [15,16]. The gear pump (model SPH0292) was used for driving the polymer solution to the spinneret. The gear pumps have a double gear motor of 30 watts power that pump the dope solution with capacity of 0.3 cm^3^/rev. The syringe pump (ISCO Model 500 D; series D) was utilized to pump the distilled water as the internal coagulant to the specific side of the spinneret. The spinneret was aligned 3 cm above the coagulation bath that exposed the outer surface of HFs to air for fractional evaporation. This setting induced the solvent and non-solvent exchange, and coagulation started on the external surface of the HFs prior to being immersed in the external coagulation bath. Three different types of membranes (M1, M2, and M3) were spun by varying the dope extrusion rate to the bore fluid injection rate as described in Table 1. Finally, asymmetric HF membranes were spun and collected onto a wind-up drum after passing the membranes through a series of rollers in the coagulation bath. After spinning, the membranes were dipped in tap water for 2 days prior to post-treatment. Table 1 represents the spinning parameters of all HF membranes.

### 2.3. Post-Treatment and Potting

Post-treatment of HFs was used to improve the performance that shrinks the pore size of the membrane. This ultimately decreases the flux rate and favors the low MWCO results of membranes. After spinning the fibers, each type of membrane was immersed in de-ionized water in a container and placed on a hot plate and gradually heated for 90 min at 90 °C [17]. The membrane was dried after the post-treatments, and then six modules of HFs were potted (i.e., 3 were not post-treated and 3 were post-treated). The characteristics of all six modules are listed in Table 2.

### 2.4. Membrane Morphology

The morphology of a cross-section of HF membrane was observed using field emission scanning electron microscopy (FESEM) via Model SUPRA 35VP, Gemini column (Phenomenex, Torrance, CA, USA). The membrane sample was snapped in liquid nitrogen and then sputter-coated with platinum and mounted onto brass plates using double-sided cellophane tapes in a vertical position [18].

### 2.5. Flux Rate and Fouling

**Figure 2:**
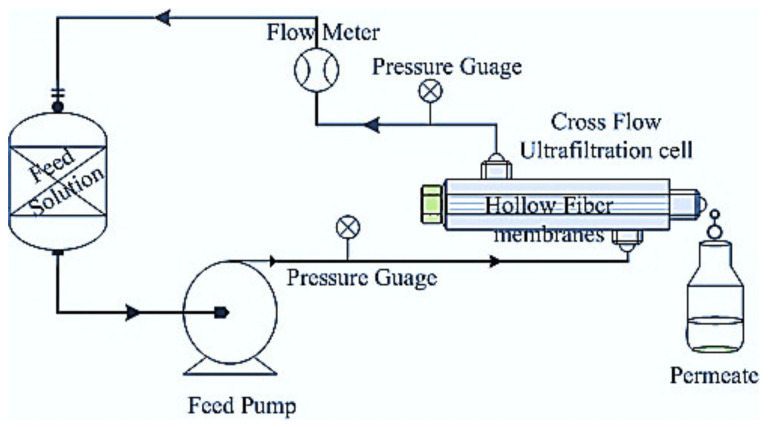
Schematic diagram of hollow fiber (out to in) cross flow filtration cell.

The performance of the untreated and post-treated HF UF membranes were evaluated in terms of pure water permeation flux (PWP) in a stainless steel cross flow test cell at 3 bar pressure, as shown in Figure 2. Distilled water was used as pure water, and HFs were placed in the steel test cell with the active skin layer facing the incoming water feed [19]. Pure water permeation (PWP) of the membranes was obtained using Equation (1):

(1) $$\mathrm{Flux}=Jw1=\frac{Q}{A\;\times \;h}=\frac{Q}{N\pi d_{o}l\;h}$$

where “*Q*” is the flow rate of permeation in liter, “*h*” is the time in hours, “*A*” is the effective membrane area (m^2^), “*d_o_*” is the outer diameter of fiber (m), “*N*” is the number of fibers, and “*l*” is the effective length of the fiber (m).

Flux recovery ratio (*R_FR_*) is an important parameter for UF processes that was determined for all fabricated membranes under the same condition as *J_w_*_1_ was calculated, and Equation (2) is used to measure the *R_FR_* [20,21]. For measuring the *R_FR_*, all HFs membranes were fouled by passing the lake water for 5–6 h. Then, the HF membranes were washed with distilled water, and water flux (*J_w_*_2_) of HFs was measured using Equation (1).

(2) $$R_{FR}\left(\%\right)=\frac{J_{w2}}{J_{w1}}\;\times \;100$$

To calculate the membrane fouling parameter, we also calculated fouling irreversible resistance using Equation (3).

(3) $$Rir=\frac{J_{w1}-J_{w2}}{J_{w1}}$$

### 2.6. Molecular Weight Cut off and Pore Size of HFs

PEG solutions of different molecular weights were used to evaluate the solute rejection profile across all post-treated and non-treated membranes. The PEG solutions (1000 ppm concentration) of 600, 1K, 3K, 6K, and 10K Daltons were used, wherein the retention/rejection rate is defined by Equation (4):

(4) $$R=\left(1-\frac{C_{p}}{C_{f}}\right)\;\times \;100\%$$

where “C_p_” and “C_f_” are the concentrations of permeate and feed, respectively.

The pore sizes of the membranes were determined through plotting solute rejection transport data against the solute diameter graph, and the Stokes radii (*r_s_*) of PEG macromolecules were calculated from their molecular weights using Equation (5) [22], wherein the symbol “*M*” represents the molecular weight of the corresponding PEG molecule. Conversely, Equation (6) can be used to determine the solute diameter (*ds*).

(5) $$r_{s}=16.73\times 10^{-10}\;M^{0.557}$$

(6) $$ds=2\;r_{s}$$

The data of the solute separation curve against solute diameter were plotted on a log normal graph to determine the mean pore size ($\mu_{\rho }$) and standard deviation ($\delta_{\rho }$) of the HFs.

### 2.7. Water Sample Collection for Metallic Ion Testing

Lake water is generally used for drinking purposes, and therefore in this study, the filtration performances of all post-treated (M1-P, M2-P, and M3-P) and non-post-treated (M1, M2, and M3) membranes against metal ion filtration were evaluated using lake water. This water was collected randomly from different sections of the Tasik Ilmu Lake in University Teknologi Malaysia.

#### 2.7.1. Color, pH, and Turbidity

A visual method was used to check the color, whereas the pH of the lake and filtered samples from all tested membranes was observed using a Hanna 211 microprocessor-based pH meter (Woonsocket, RI, USA).

A turbidity meter is normally utilized to check the water clarity in drinking water samples. Thus, in this study, the Turbid meter (Thermo Corporation Orion Aquafast^™^ II, Waltham, MA, USA) was used to measure the turbidity of water samples obtained from the lake and after its filtration via all formulated membranes. The Turbid meter works on the nephelometric and radiometric principles.

#### 2.7.2. Chlorides (Cl^−1^)

A Hanna HI 701 instrument with NN diethyl-p-phenylenediamene reagent was used for the quantitative estimation of chloride ions from the lake and filtered water samples. This instrument has the range of 0.00 to 2.50 ppm with an accuracy ±0.03 and worked on the USEPA method 330.5.

#### 2.7.3. Metal Ions

An inductively coupled plasma–mass spectrometer (ICP-MS) (Perkin Elmer, Waltham, MA, USA) was used for the determination of different metal ions: Mg^+2^, K^+^, Cr^+2^, As^+3^, Na^+1^, and Fe^+2^ ions in the lake and filtered water samples via different fabricated and post-treated membranes. All the water samples were collected in polyethylene containers that had been previously rinsed and washed with a 10% solution of nitric acid. Before testing with ICP-MS, 60% nitric acid was added into the sample for stabilization.

## 3. Results and Discussion

Idris et al. reported in detail the effect of LiBr on the performance of PES HFs in terms of flux rate, hydrophilicity, MWCO, and pore size, and the authors concluded that 3 wt. % of LiBr in the formulation provided the best results in terms of MWCO and 2.83 KDa with high flux rate [14,23]. In this work, all HFs were fabricated using the same 3% LiBr with PES polymer for the filtration of metal ions from lake water. The lake water was suitable for irrigation purposes and belongs to class 1/II [23].

### 3.1. Post-Treatment, Membrane Dimension, and Fiber Morphology 

**Figure 3:**
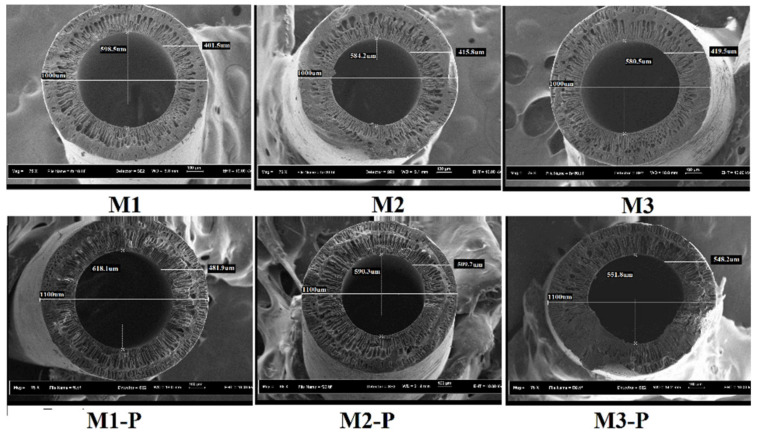
The FESEM cross-section results with dimension calculations (i.e., outer diameter, wall thickness, and inner diameter in nanometers).

The presence and suitable amount of non-solvents in the polymer solution played a positive effect that boosted the formation of finger-like macro-voids and suppressed the spongy entities on the HF membrane [24]. During the spinning process, the ratio of DER to BFIR was varied from 2.25, 2.50, and 3.0, in which flow of BFR was constant (5 mL/min), whereas DER was changed and controlled via the digital gear pump, while others parameters persisted in the same way (see Table 1). At different dope flow rates, the velocity, shear stress, and shear rate distributions of the polymer solution are different inside the spinneret. Conversely, sheer rate is greatly increased through increasing the flow rate of the dope solution [25]. Inside the spinneret, the polymer solution is under stress. When the polymer solution comes out of the spinneret in the form of HFs, the stress is released perpendicular to the fiber axis, which will result in the expansion of the fiber diameter. However, the axis of HFs also suffers from parallel stress that will remain until the fiber reaches the gelatin bath. The release of parallel stress will elongate the fiber and decrease the fiber diameter [14]. In the present study, changing the DER affected the internal diameter (ID) of the central fiber hole and wall thickness (WT) of the HF membrane. In the non-treated membranes, a higher amount of DER decreased the ID, whereas it enhanced the WT of membranes M1 to M3, as shown in Table 3 and Figure 3. Due to the higher DER, the M3 and M2 had higher stress inside the spinneret than M1, which was minimized by pushing the ID toward its center (tended to decrease the ID) in the corresponding HFs. This inward push of polymer solution was responsible in M3 and M2 membranes for reducing the size of internal holes (and increasing the WT of HFs other than M1 (see Table 3)). Moreover, M2 and M3 membranes contained slightly less finger-like entities beneath the outer skin than M1.

**Figure 4:**
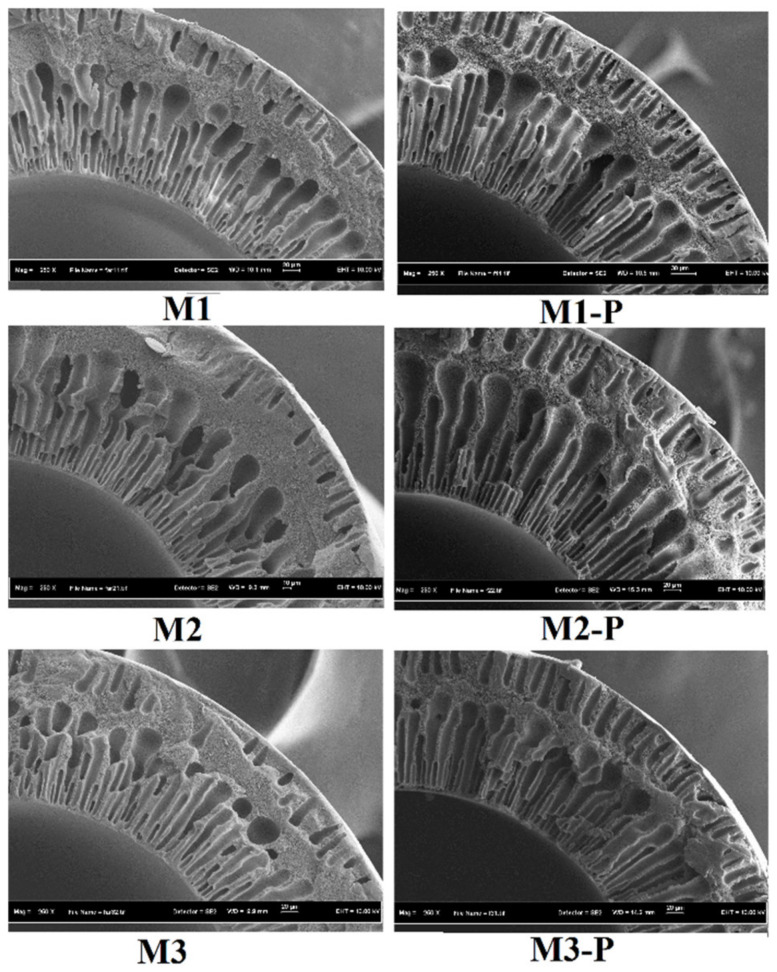
Cross-sectional images of side wall of non-post-treated (**M1**, **M2**, and **M3**) and post-treated (**M1-P**, **M2-P**, and **M3-P**) HF membranes, obtained by FESEM.

After the post-treatment, all the HFs were swelled (internally and externally), and the size of outer diameter (OD) was enhanced by nearly 100 um. The swelling process of HFs also reduced the size of its central hole, whose reduction seemed proportional to the increasing amount of DER. After the post-treatment, membranes M1, M2, and M3 showed 3.66, 4.76, and 7.89% reduction of the central hole, and wall thickness increased up to 80.4 um, 93.9 um, and 128.7 um, respectively. Figure 4 represents the FESEM images of cross-sections of side wall of post-treated and non-post-treated HF membranes. In this figure, formation of double skins is clearly shown, which contained a common finger-like entity with central sponge-like assembly. Generally, fast coagulation processes favor this kind of structure during immediate dope contact with the coagulant. Ismail et al., [5] also reported the presence of double skins in their HFs, which influenced the molecular orientation of the fiber and affected the membrane separation performance.

After the post-treatments with hot water, the size of the sponge-like layer was reduced in the middle and the finger-like structure elongated and extended through the sponge-structured area. The overall morphology of all post-treated membranes looked similar, but these membranes showed different pore sizes and MWCO results with different metal ion rejection rates (as mentioned in Section 3.2 and Section 3.5).

### 3.2. MWCO and Pore Size

**Figure 5:**
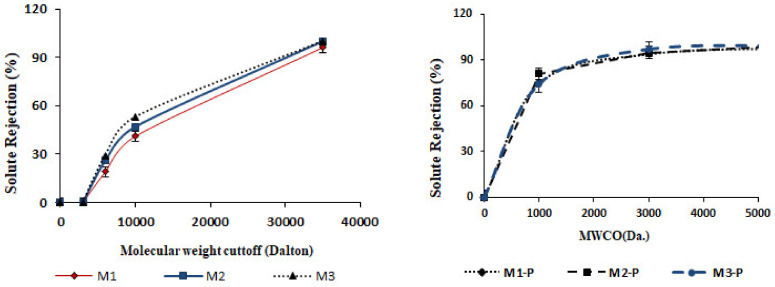
The MWCO profile of all formulated membranes (SD ± 3%).

The MWCO and pore size of the HFs was obtained from ultrafiltration (UF) experiments using PEG solutions of different molecular weights. Figure 5 represents the MWCO results, wherein solute rejection percentage was drawn against PEG solutions. Table 4 shows the mean pore size ($\mu_{\rho }$) and standard deviation ($\delta_{\rho }$) of the HFs calculated by log normal graph, whose detailed method is explained in Section 2.7. An increment in the DER from 11.35, 12.5, and 15.6 mL/min increased the wall thickness of HFs to 401.5, 415.8, and 419.5 um, respectively (see Table 3). This increment in wall thickness increased the sieving area of solutions inside the HFs and decreased the pore size, influencing the solute separation results. Table 4 shows that pore size of M1 to M3 membranes was decreased from 3.85 to 3.59 nm, with a corresponding decrement in MWCO from 34 to 31 KDa.

Barzin et al. [14] fabricated PES/PVP-based HF membrane and post-treated the fiber in hot water at 95 °C for 30 min. The authors reported that after post-treatments, dimension was unchanged, and MWCO slightly increased. However, in the current case, after the post-treatment, dimension of HFs was changed, the size of the central hole was reduced, and wall thickness increased due to swelling phenomena, which reduced the pore size and MWCO. Prior to the post-treatment, MWCO was observed up to 34–31 kDa. This was greatly reduced to 2.2–2.1 kDa. These results were opposite to those of the findings of Barzin et al. [14], which might have been due to the presence of inorganic salt additives (LiBr) in casting solutions in the formulation.

### 3.3. Effects of DER on Membrane Flux and Its Anti-Fouling Properties

**Figure 6:**
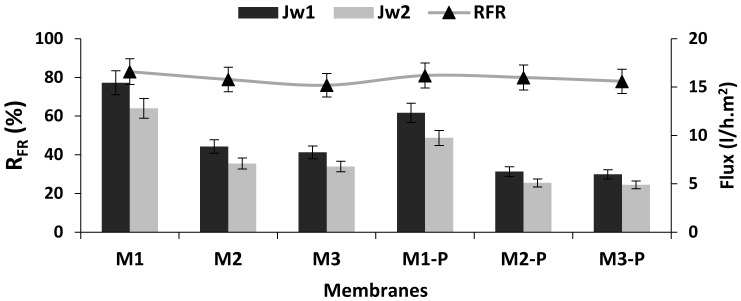
Flux rates and flux recovery ratio of hollow fiber membranes.

**Figure 7:**
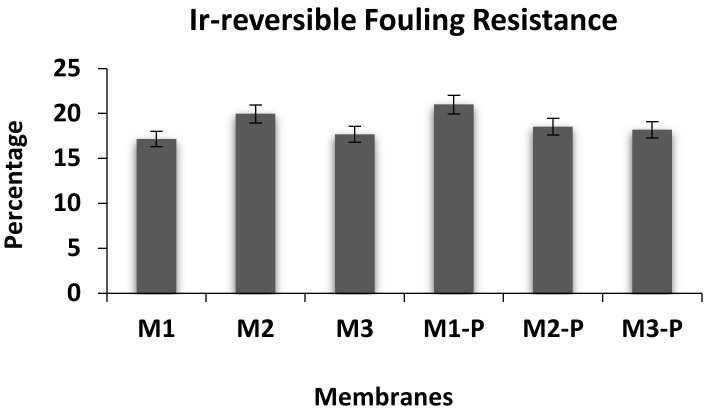
Fouling resistance curve of all membranes.

Figure 6 shows the results of flux rate (*J_w_*_1_ and *J_w_*_2_) and flux recovery ratio, whereas Figure 7 represents the irreversible fouling resistance results. M1 and M1-P membranes showed the highest flux rate in non-post-treated and post-treated membrane groups, respectively. These higher flux rates might have been due to their higher internal hole of fiber diameter (ID) and lesser wall thickness, as described in Table 3 and Figure 3. M1 membrane also showed minimum irreversible resistance (Figure 7) and exhibited a comparably good and dense capillary system (Figure 3).

As shown in Figure 6, when the dope extrusion rate increased, the PWF of the HF membranes decreased, and the separation performance for low-molecular-weight PEG solutes was improved (Figure 5). This means that the outer skin layer of the HFs also became thicker with the increasing dope extrusion rate (Figure 3). This result is consistent with the observed pore-sized, MWCO and the membrane morphology because the spun fiber with higher shear had higher resistance for water permeation [5].

After the post-treatments, the flux rate of M1-P was slightly reduced in comparison with M1 membranes, but its solute separation performance was 93.4% improved (Table 4). Although the membranes M2 and M3 spun with 12.5 and 15.6 mL/min DER, they showed very little difference in their PWP results, which seemed similar to the findings of Qin et al., Qin and his co-workers reported that after a certain critical value of DER, the PWP and separation ability for a specific solute of the spun fibers were not changed dramatically [26].

The antifouling enactment (Figure 6) of all formulated membranes was evaluated by the flux recovery ratio (*R_FR_*) and irreversible fouling resistance graph (Figure 7). Since the purpose of the research was to produce an excellent membrane to convert raw lake water into drinking water, we fouled the membrane by raw lake water for 5 to 6 h. The second measurement of the flux rate (*J_w_*_2_) of the PWP decreased up to 17–22% in all tested membranes. This result was due to the fouling induced by the deposition and adsorption of the impurities of lake water (algae, clay, bacteria, etc.) onto the membrane surface. After washing the HFs with distilled water, more than 80% *R_FR_* was achieved. Further, membrane M1 exhibited noticeable low irreversible fouling. These results represent the outstanding behavior of HF membrane towards *R_FR_* for the cleaning of lake water for longer periods of time. Membrane M1 and its post-treated option M1-P showed the best values of *R_FR_* (83 and 81%), which may have been due to its high resistance to the impurities because of lesser wall thickness and a larger central hole of HFs than the remaining formulated membranes.

### 3.4. pH, Color, and Turbidity

The pH value of filtered and non-filtered lake water was marginally changed (6.5–7.20), which showed that all types of fabricated HFs had no influence on the pH of lake water. However, the pH value of permeate and feed was under class I and thus in the acceptable range for drinking water [23].

**Figure 8:**
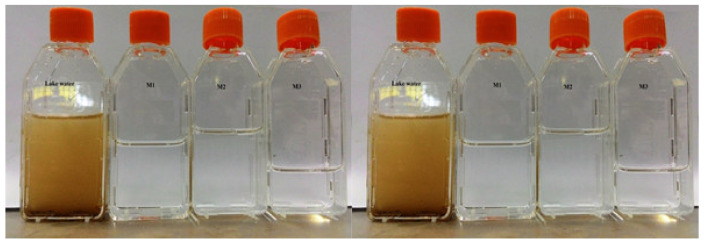
The visual result of change in color of lake water via non-treated and post-treated membrane after filteration.

Figure 8 represents the visual results of filtration of lake water through all the tested membranes. After filtration, it was clearly observed that the light brown color of the water and the suspended particles (such as mud, algae, etc.) were totally removed. The electrostatic repulsion between the membrane materials and the color molecules of feed solution (lake water) and the pore size of the HFs membranes were mainly responsible for the degree of color removal [27]. Since the size of most of the dissolved solids and particles in the feed solution was smaller than the pore size of the formulated HFs (Table 3), this proposes the idea that electrostatic repulsion was primarily responsible for the color removal ability for all post- and non-post-treated membranes.

**Figure 9:**
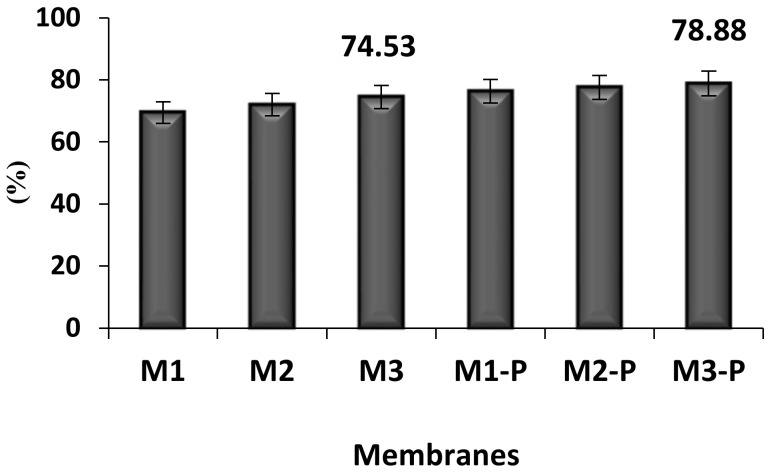
Turbidity clearance percentage of all tested HF membranes after filtration of lake water (*n* = 3).

In the water treatment system, UF application is considered a good tool to reduce the turbidity of feed solutions. The suspended solids such as silt, clay, and sand are responsible for the high turbidity result [28]. As these suspended particles are larger than the dissolved metal ions or other ions, they are hence removed through the pore size of HF membranes during the UF filtration process. Figure 9 represents the turbidity results that showed that higher DER increased the wall thickness of HFs. This reduced the pore size of the membranes, which ultimately provided the best result for turbidity removal. In the groups of non-post-treated and post-treated membranes, M3 and M3-P membranes, respectively, were spun with the higher rate of DER, showing higher percentage removal of turbidity (74.53% and 78.9%, respectively).

### 3.5. Metals Ions

Generally, polymer-enhanced (PE) UF and micellar-enhanced (ME) UF are the common methods utilized to obtain high metal ion removal, and simple UF membrane is not able to eliminate metal ions since the pore size of the UF membrane is significantly larger than the dissolved metal ions. In the ME ultrafiltration system, a specific quantity of surfactant is used that acts as the ions binder, which aggregates into micelles and binds with the metal ions to produce a larger micelle. On the other hand, in PE ultrafiltration methods, water-soluble polymers are used, which bind with metal ions and form a macromolecule that cannot pass via the HFs because of its bigger molecular size in comparison to the pore size of membranes [29]. In this experiment, the lake water was directly exposed to the HF membranes, and none of these two systems were applied.

**Figure 10:**
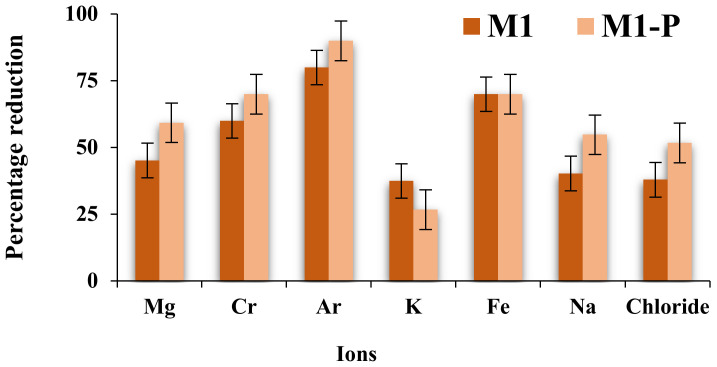

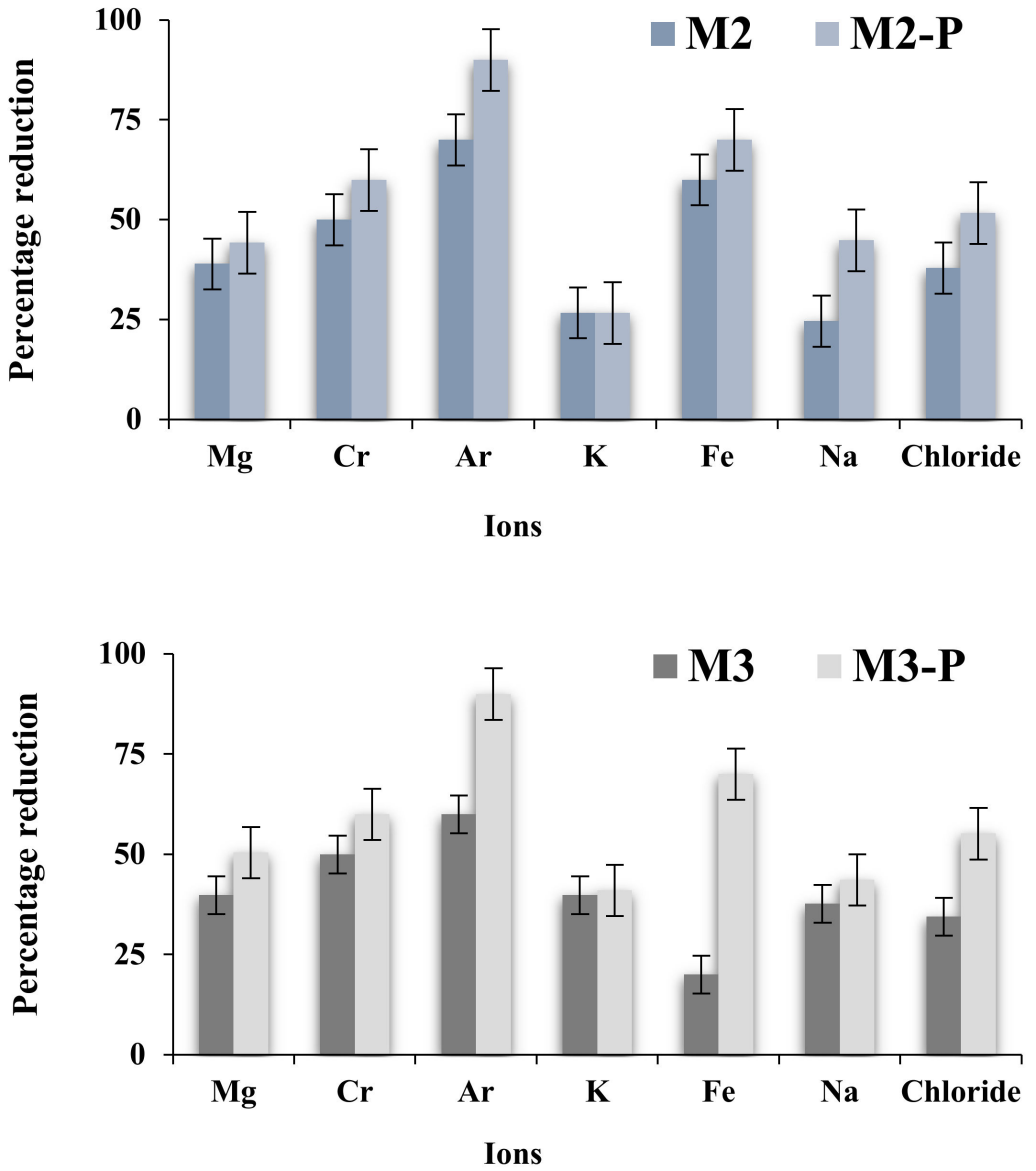
The weight percentage of metal ion rejection after the filtration of lake water through all fabricated and post-treated membranes (SD ≤ 5%).

In this study, we used lake water as a feeding source of contaminated water that contained different metal ions in very low concentrations (i.e., Mg^+2^, Cr^+2^, Ar^+2^, Fe^+2^, and Na^+^ with concentrations of 0.01, 0.001, 2.51, 0.1, and 5.04 mg/L). Since a suitable percentage of salt in drinking water is also a key point, filtration of chloride ions (Cl^−^, 0.29 mg/L concentrations) was also considered. Figure 10 represents the percentage reduction of metal and chloride ions after filtration from all fabricated membranes. The results obtained from ICP-MS showed that the PES/LiBr membrane effectively removed the dissolved metals with a high permeation and rejection rate, although metals were present in very low quantity. As we mentioned earlier, the size of metal ions are significantly lower than the pore size of the membranes, and therefore it was believed that the internal orientation of the membrane layers (sponge-like and finger-like entities) and electrostatic repulsion were mainly responsible for removal of dissolved metal ions. The presence of inorganic additives (LiBr) could have involved the electrostatic repulsion because on the membrane surface, LiBr can exist as Li^+1^ and Br^−1^, which might repel the incoming metal ions, essentially passing through them with the lake water. This has been observed, although there was not much difference between M2 and M3 membranes in terms of pore size, morphology, and MWCO, but due to different DER, they exerted different electrostatic repulsions on metal ions. The M3 membranes showed 55.36% (as an average) clearance of metal ions, which was 14.10% higher than M2. The metal removal ability is efficiently enhanced by the post-treatment process, especially for M1-P and M2-P membranes. Membranes M1-P showed more than 60% (an average of all) metal removal efficiency, in which Ar^+2^ showed 90% clearance rate, whereas Cr^+2^ and Fe^+2^ showed 70% each.

Thus, it was sturdily believed that due to difference in DER, the process of post-treatments shrank the pore size, reduced the MWCO, and increased the wall thickness of HFs membranes; further, LiBr in membrane composition enhanced the repulsive interaction with the dissolved metallic ions.

## 4. Conclusions

Three different types of HF membranes were successfully fabricated with 2.25, 2.5, and 3.1 ratios of DER to BFR and then were treated with hot water bath for post-treatments.

Thickness of HFs increased with an increasing amount of DER and were further enhanced by the post-treatment process. These changes affected membrane morphology, water permeation rate, pore size, and MWCO of all fabricated membranes. Results showed that the rejection rate of PEG solute increased with an increase in dope extrusion rate that might have been due to denser fiber wall thickness. Moreover, after the post-treatment, the MWCO of HFs reduced from 34 kDa to 2. kDa, with slight reduction in flux rate (15.45 to 12.34 L/h.m^2^), especially in the case of M1 membranes. Once the DER reached a certain value (critical point), the rejection of PEG solutes and water permeation became approximately equal, which was observed especially in the case of M2 and M3 membranes. This was probably due to the development of less tightened skin structure at high DER in the HFs, as shown by FESEM pictures (Figure 3).

Lake water is an important drinking source, and certain metal ions such as arsenic and chromium are some of the most dangerous contaminants. The existence of LiBr inorganic salt in the membrane surface formed lithium and bromide ionic structures that might exert some kind of electrostatic repulsion on the incoming metal ions in the raw lake water, thus enabling the removal of metals ions effectively. Membrane M1-P showed an outstanding and high rejection rate for the filtration of most of the tested metal ions in comparison with other HFs. The M1-P rejected most metals ions (Mg^+2^, Cr^+2^, Ar^+2^, Fe^+2^, and Na^+^ ions), with a more than 50% rejection ratio, in which chromium and iron were rejected by up to 70% and arsenic by up to 90%. This higher type of rejection with some acceptable flux rate was quite difficult as compared to nano-filtration and other filtration techniques. Moreover, after the filtration process, the concentrations of the metal ions were in the permissible drinking water range as per the published list of the World Health Organization (WHO) [23].

## Figures and Tables

**Table 1:** Spinning conditions of hollow fiber membranes.

Spinning Parameters	Membranes		
	M1	M2	M3
Bore fluid injection rate (mL/min)	5	5	5
Dope extrusion rate (mL/min)	11.35	12.5	15.6
Ratio of dope extrusion rate and bore fluid injection	2.25	2.5	3.1
Fiber take-up roller radius	0.32 m	0.32 m	0.32 m
Fiber take-up speed (rev/min)	4.5	4.5	4.5
Air gap between spinneret and coagulant bath	3.5 cm	3.5 cm	3.5 cm
External coagulation bath	Tap water	Tap water	Tap water
External coagulation bath Temp.	10 °C	10 °C	10 °C

**Table 2:** Geometrical characteristics of HFs and modules.

Membranes	M1	M2	M3	M1-P	M2-P	M3-P
**No. of fibers**	20	20	20	20	20	20
**Fiber outer diameter (cm)**	0.100	0.100	0.100	0.110	0.110	0.110
**Fiber inner diameter (water bore dia. (cm))**	0.0599	0.0584	0.0581	0.0618	0.0590	0.0552
**Total permeation surface area of HFs (cm) ^2^**	0.9677	0.9813	0.9452	1.2071	1.2991	1.3180

**Table 3:** The dimension of fabricated and post-treated HFs membranes, measured by FESEM standard software, which included the outer diameter (OD), inner hole diameter (ID), and wall thickness (WT).

Memb. #	OD (um)	ID (um)	WT (um)	Ratio of OD with	
				ID (%)	WT (%)
**M1**	1000	598.5	401.5	59.85	40.15
**M2**	1000	584.2	415.8	58.42	41.58
**M3**	1000	580.5	419.5	58.05	41.95
**M1-P**	1100	618.1	481.9	56.19	43.81
**M2-P**	1100	590.3	509.7	53.66	46.34
**M3-P**	1100	551.8	548.2	50.16	49.84

**Table 4:** The pore size ($\mu_{\rho }$), standard deviation ($\delta_{\rho }$), and MWCO profile of post-treated and non-post-treated membranes.

Memb. #	*µ_p_* (nm)	*δ_p_* (nm)	MWCO (KDalton)
**M1**	3.59	1.48	≅34
**M2**	3.72	1.45	≅33
**M3**	3.85	1.64	≅31
**M1-P**	2.90	2.15	≅2.2
**M2-P**	2.83	2.12	≅2.2
**M3-P**	2.75	2.10	≅2.1
